# Engagement Promotes Abstinence in a Web-based Cessation Intervention: Cohort Study

**DOI:** 10.2196/jmir.2277

**Published:** 2013-01-28

**Authors:** Amanda Richardson, Amanda L Graham, Nathan Cobb, Haijun Xiao, Aaron Mushro, David Abrams, Donna Vallone

**Affiliations:** ^1^Department of Research and EvaluationLegacyWashington, DCUnited States; ^2^Department of Health, Behavior and SocietyJohns Hopkins University School of Public HealthBaltimore, MDUnited States; ^3^Schroeder Institute for Tobacco Research and Policy StudiesLegacyWashington, DCUnited States; ^4^Lombardi Comprehensive Cancer CenterGeorgetown University Medical CenterWashington, DCUnited States; ^5^Department of MarketingLegacyWashington, DCUnited States

**Keywords:** Internet, smoking cessation, intervention studies, use effectiveness

## Abstract

**Background:**

Web-based smoking cessation interventions can have a public health impact because they are both effective in promoting cessation and can reach large numbers of smokers in a cost-efficient manner. Their potential impact, however, has not been realized. It is still unclear how such interventions promote cessation, who benefits most, and how to improve their population impact.

**Objective:**

To examine the effectiveness of a highly promoted Web-based smoking cessation intervention to promote quit behavior over time, identify the most effective features, and understand who is most likely to use those features by using unweighted and weighted analyses to estimate the impact in the broader pool of registered site users.

**Methods:**

A sample of 1033 new adult registrants was recruited from a Web-based smoking cessation intervention by using an automated study management system. Abstinence was assessed by self-report through a mixed-mode follow-up (online survey with telephone follow-up for nonrespondents) at 1, 3, and 6 months. Software tracked respondents’ online activity. Generalized estimating equations (GEE) were used to examine predictors of website utilization and how utilization promoted abstinence using unweighted and weighted data.

**Results:**

The 7-day point prevalence abstinence rates at 6 months ranged from 20.68% to 11.13% in the responder and intent-to-treat samples, respectively. Predictors of abstinence in unweighted analyses included number of visits to the website as well as accessing specific interactive or engaging features. In weighted analyses, only number of visits was predictive of abstinence. Motivation to quit was a key predictor of website utilization, whereas negative partner support decreased the likelihood of increasing visits or accessing engaging features.

**Conclusions:**

Engagement is critical to promoting smoking cessation. The next generation of Web-based smoking cessation interventions needs to maximize the initial engagement of all new visitors and work to retain those smokers who proceed to register on the site.

## Introduction

Despite a substantial decline in smoking prevalence over the past 40 years, nearly 1 in 5 adults (45.3 million) in the United States still smokes, and the rate of prevalence reduction has slowed in the last decade [1]. Most smokers (approximately 70%) say that they want to quit, and each year roughly one-half of smokers try to quit, but less than 6% succeed [2]. Furthermore, only 31.7% have used counseling and/or medications when they tried to quit [2], highlighting the need for cost-effective, engaging, and sustainable cessation interventions that can reach and motivate smokers to quit and help them remain abstinent. Given the substantial influence of the Web, with its over 2.67 billion users across the globe [3] and its increasing use as a mechanism to seek health information [4,5], Web-based smoking cessation interventions show significant potential to promote cessation if optimized to be maximally effective (a product of reach and efficacy divided by cost) [6,7].

The 2008 Clinical Practice Guidelines for Treating Tobacco Dependence [8] assert that Web-based interventions are a “highly promising delivery system for tobacco dependence treatment.” These interventions can be built to run on multiple platforms including tablets and mobile devices [9-11], are easily accessible and available all hours of the day, provide anonymity to those smokers who prefer privacy [12], and remove the logistical and financial barriers associated with some pharmaceutical and/or in-person behavioral counseling interventions. Additionally, Web-based interventions have evolved to engage smokers with interactive elements including multimedia content, social networks, blogs, forums, and virtual communities [9,13-15]. Web-based interventions show evidence of effectiveness in adult tobacco users [16-18], with recent randomized trials reporting quit rates as high as 26% at 6 months posttreatment [19-21]. Although several studies have documented increased rates of cessation associated with tailoring [14,22-27], greater website exposure [25,28-36], and a supportive vs information-only content [24,33,34], a recent review concluded that the strength of the evidence for effectiveness is only moderate and could benefit from additional research to improve population impact [37]. This review also concluded that there is currently insufficient evidence for the effectiveness of multicomponent, interactive, or additional components, such as bulletin boards, in promoting cessation [37]. Consistent with the National Institutes of Health’s strategic priorities, there is an extraordinary opportunity to conduct more rigorous implementation/dissemination research to improve the real-world impact of Web-based interventions for smoking cessation [38-40].

Tobacco control simulation models suggest that Web-based interventions have extraordinary potential to contribute to a further decrease in population-level smoking rates if effective and if promoted sufficiently [41]. For the full potential of these interventions to be reached, however, further research is needed to clarify the degree to which Web-based interventions are effective, how and why these interventions work, the factors influencing their effectiveness, and their long-term benefits in a real-world setting.

The purpose of this study was to build upon previous literature to examine the effectiveness of an interactive Web-based cessation intervention, BecomeAnEX.org [42], in promoting quit attempts and abstinence over a 6-month period in a large sample of newly registered users. Specific aims of the study were to (1) identify whether increased use of the website promotes quit attempts and abstinence, (2) determine which components of the program are most effective in promoting cessation, and (3) examine the demographic characteristics, psychosocial factors, and smoking behaviors associated with greater use of the program and its associated features. We hypothesized that increased utilization of the website and accessing the BecomeAnEX.org community would be associated with quit behavior over the course of this study.

## Methods

### The BecomeAnEX.org Intervention

**Figure 1:**
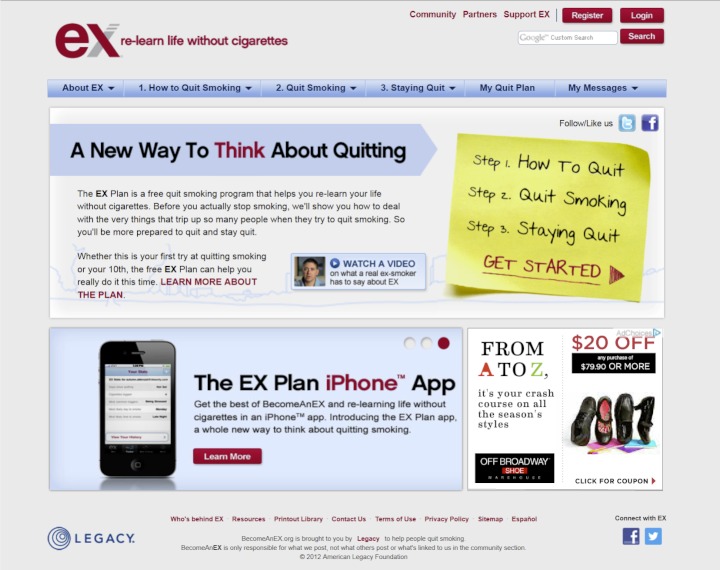
Screenshot of the home page of the BecomeAnEX.org website.

**Figure 2:**
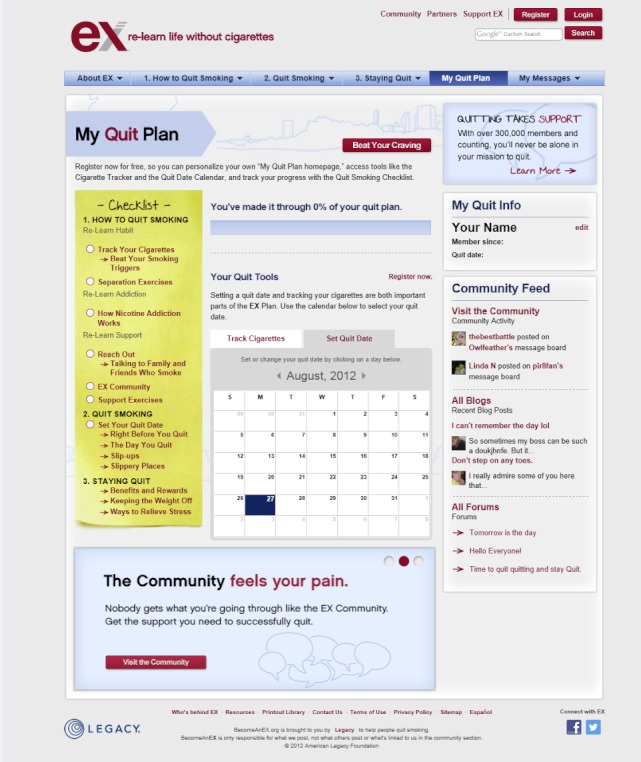
Screenshot of the My Quit Plan page of the BecomeAnEX.org website.

BecomeAnEX.org is a free, branded, evidence-based intervention developed in accordance with the 2008 US Department of Health and Human Service’s Clinical Practice Guidelines [8]. The site was developed by Legacy, a nonprofit organization that develops smoking prevention and cessation programs, in collaboration with the Mayo Clinic Nicotine Dependence Center. The overarching goal of the site is to educate smokers wanting to quit and provide the tools necessary to support their quitting efforts. The site provides smokers with detailed information and action steps to help them “re-learn life without cigarettes,” in part by disassociating smoking from common daily activities that would otherwise function as smoking cues, such as driving or drinking coffee. BecomeAnEX.org is designed to support smokers through their journey of quitting smoking and preventing relapse. BecomeAnEX.org is available on multiple platforms, including mobile, and is designed to engage smokers through the following core elements: (1) My Quit Plan, a checklist that helps smokers set a quit date and tracks their progress through the steps of the quit plan; (2) a Cigarette Tracker to help smokers identify and track their smoking triggers; (3) Beat Your Smoking Triggers, a list of common triggers with tips for dissociating cigarettes from them; (4) separation exercises to teach smokers how to separate a cigarette from its trigger; (5) support exercises to help smokers identify who they can turn to for support when quitting smoking; (6) Quit Smoking Resources, a list of national organizations, state quitlines, and websites that smokers can turn to for additional information and help to quit smoking; (7) text and videos detailing the importance of medication and the different types available; and (8) BecomeAnEX.org Community, a large online network of current and former smokers. See Figures 1 and 2 for screenshots of the website.

### Study Participants and Enrollment

**Figure 3:**
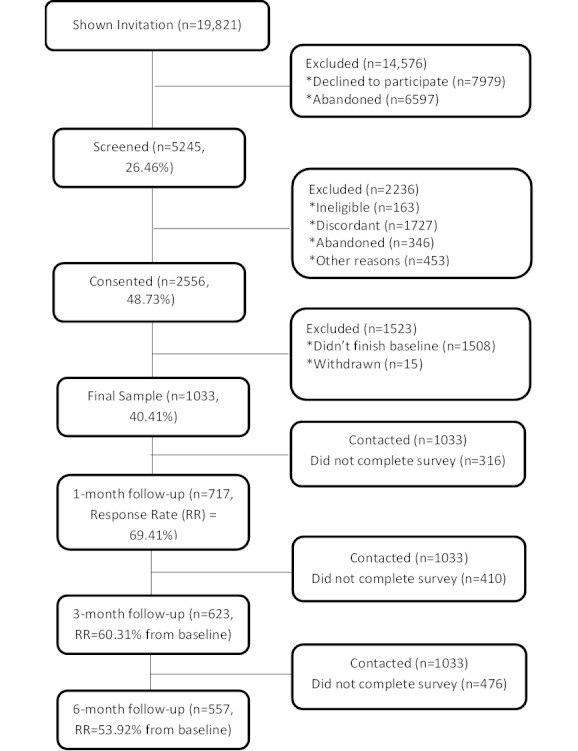
CONSORT diagram of study recruitment.

This was a prospective, observational study of a cohort of participants recruited via an automated study management system at the time of enrollment on BecomeAnEX.org. To be eligible, BecomeAnEX.org registrants had to be current smokers, age 18 years or older, and willing to provide a valid email address and telephone number. From January 19 through May 23, 2011, a total of 19,821 individuals were invited to participate in the study (Figure 3).

Enrollment for certain demographic subgroups (eg, white females) was deactivated once targeted enrollment goals were met. The invitation described the terms of the study, expectations regarding follow-up assessments, and the availability of incentives for completion of follow-up surveys. Registrants who accepted the invitation (n=5245) completed online eligibility verification and online informed consent. Those who were eligible (current smokers, age ≥18 years, provided valid email/telephone number) and consented (n=2556) were sent an email with a link to the baseline survey that they had to complete within 24 hours. A total of 1048 individuals completed the baseline survey; of those, 15 were subsequently withdrawn because of duplication in study registration, leaving an effective sample size of 1033. Respondents were then contacted for follow-up surveys at 1, 3, and 6 months after enrollment. Intensive email and telephone follow-up yielded response rates of 69.41% (717/1033), 60.31% (623/1033), and 53.92% (557/1033), respectively.

### Data Collection

Data for the study were obtained through 2 sources: (1) self-report assessments completed at baseline and at 1, 3, and 6 months after enrollment, and (2) online tracking software. At each follow-up, respondents were sent an email offering an incentive of US $25 to complete an online follow-up survey. Reminder emails were sent to participants 3 and 7 days after the initial email. Attempts were made to contact nonrespondents by phone to complete the follow-up survey starting at 10 days after the initial email. At least 15 but no more than 20 attempts were made to contact nonrespondents over multiple days at different times of day. Participants were reimbursed US $15 for completing the follow-up assessment by phone. The lower reimbursement offered through the telephone follow-up was intended to encourage online follow-up. Individual-level tracking metrics of BecomeAnEX.org utilization (eg, number of log-ins, pages viewed) were recorded by using Omniture SiteCatalyst software [43] and transferred to a relational database linked to the automated study management system. Utilization of BecomeAnEX.org via mobile devices was not recorded. At the time of the study, the mobile site associated with BecomeAnEx.org was a limited version that contained only a portion of the functionality of the full site and represented only anonymous use (ie, there was no log-in required or available). As such, although use of the mobile site was tracked by Omniture SiteCatalyst, it was not possible to record individual use by study participants. Websites other than BecomeAnEX.org were not monitored. This study received Institutional Review Board (IRB) approval by Schulman Associates IRB (previously Independent IRB, Inc).

### Weighting

Data obtained on the study sample were poststratified to be representative of the larger population of new registrants to BecomeAnEX.org during the period of study recruitment and adjusted for nonresponse. Variables on which the study sample were weighted include gender, age, education, and race/ethnicity of new registrants. Each of these demographic variables was significantly correlated (*P*<.05) with baseline levels of the smoking variables mentioned previously (eg, motivation to quit, quit attempts in past year, and cigarettes per day). Demographic information was also significantly correlated with website usage during the study period.

### Measures

The main outcomes were self-reported number of quit attempts, 7-day abstinence, and 30-day abstinence measured at each of the 3 follow-ups. Self-report of smoking behavior is commonly accepted in national Web-based cessation trials [19,20,22,23,29,30,44] when biochemical verification is neither feasible nor necessary [45]. Quit attempts were assessed with the question: “Since our last survey, how many times have you intentionally quit smoking (not even a puff) for at least 24 hours?” Abstinence at 7 and 30 days was measured at each follow-up with the question: “About how long has it been since you completely stopped smoking cigarettes?”

Main independent variables to measure website “dose” were 3 Omniture SiteCatalyst metrics recorded at each time period: (1) the number of visits to BecomeAnEX.org, (2) the number of minutes on the website, and (3) the number of pages accessed on the website. All exposure metrics recorded represent unmodified industry standards preset in Omniture SiteCatalyst. Specifically, time online was calculated as the duration between the first page view and last page view of a session, whereas a session times out after 30 minutes of inactivity. Cumulative values for each of these metrics, as measured by Omniture SiteCatalyst, were recorded at each follow-up survey. Additionally, data on the features accessed by the respondents (detailed in the Intervention section) were collected and examined. The top 5 most commonly accessed BecomeAnEX.org website features or pages within the study sample were examined in relation to quit behavior, namely: (1) My Quit Plan, (2) the BecomeAnEX.org Community, (3) separation exercises, (4) Beat Your Smoking Triggers, and (5) the “set-a-quit-date” feature. Usage of these features was coded categorically with 3 levels: never accessed, accessed once, and accessed 2 or more times. Additional metrics of interest were self-reported use of the BecomeAnEX.org Facebook and Twitter accounts that encouraged use of and provided links to the full site.

Control variables included age, race/ethnicity (non-Hispanic white, non-Hispanic black, Hispanic, and other), and education, which was categorized as less than a high school diploma, high school diploma or GED (General Educational Development) test, 1-3 years of college, and college degree (or higher). Baseline smoking variables that may potentially be related to both website utilization and quit behavior were also measured, including motivation to quit as measured by the question: “On a scale of 1 to 10, how much do you want to quit smoking?”, number of quit attempts in the past year, nicotine dependence as assessed by the Fagerstrom Test for Nicotine Dependence [46], peer smoking (as measured by the question “Think about the 5 people outside of your family that you spend the most time with. How many of them smoke?”), household smoking (yes/no), partner smoking status (yes/no), and past year use of at least 1 cessation aide (eg, pharmacotherapy). The following psychosocial variables were measured at baseline given their association to quit behavior: social support as measured by a modified 6-item version of the Partner Interaction Questionnaire (PIQ) [47], health status (excellent, very good, good, fair, poor), advice to quit from a health care provider (yes/no), sadness as measured by the 12-item Short Form Health Survey (SF-12) [48] on a 6-point scale (with the question “How much of the time during the past 4 weeks have you felt downhearted and blue?”), perceived stress as measured by Cohen’s Perceived Stress Scale [49], and frequency of Internet use.

### Analysis

Data on the study cohort were analyzed in comparison to all other new registrants on the website during the period of study recruitment to assess differences between the study population and the more general pool of registrants. Registration data included demographic information and several questions assessing smoking behaviors, including cigarettes per day, motivation to quit, and quit attempts in the past year.

Both unweighted and weighted analyses of primary outcomes were completed. First, frequencies and descriptive data were calculated for baseline demographic characteristics, smoking behaviors, and psychosocial factors. Next, the median and interquartile range (IQR) of each of the utilization metrics and the frequency of utilization of each of the BecomeAnEX.org features were calculated. Smoking behavior at each follow-up survey was calculated by using self-reported smoking status. Abstinence rates were calculated 2 ways: (1) responder-only analyses that only considered participants at each follow-up with complete data, and (2) intent-to-treat (ITT) analyses that considered all study participants and counted participants lost to follow-up as smokers. Abstinence was also calculated as a function of the number of visits to the website.

Logistic regression analyses by using generalized estimating equations (GEE) were used to examine the association over time between website use and quit behavior [50]. Briefly, GEE accounts for correlations in repeated measurements and produces efficient regression parameter estimates and accurate standard errors [51]. Two models were calculated. The first model estimated the association between website usage variables (eg, number of visits) and quit behavior, adjusting for potentially important confounding variables mentioned in the Measures section. Because of the skew of the website usage variables, each was log-transformed. Since visits to the website were highly correlated with number of minutes on the website (*r*=0.84, *P*<.001) and number of pages accessed on the website (*r*=0.78, *P*<.001), results are presented only for number of visits. The second model adds in 4 of the top 5 Web features or pages accessed by participants and coded usage categorically into 3 levels: never accessed, accessed once, and accessed 2 or more times. Although My Quit Plan was the most-accessed page, it was removed from the analysis because of the low variability in its use (accessed by approximately 90% of the sample) and because it encompasses and provides links to the other features of the site. An additional set of models examined predictors of number of visits to the website as well as usage of the 2 features of BecomeAnEX.org that were significantly associated with abstinence, namely the BecomeAnEX.org community and separation exercises. These models included all the control variables described previously.

## Results

### Unweighted Data

The demographic characteristics of study participants are displayed in Table 1. There was a roughly even split between males (48.31%, 499/1033) and females (51.69%, 534/1033) and most participants were 25 to 49 years of age (85.38%, 882/1033). Consistent with our targeted recruitment goals, there was good representation of non-Hispanic blacks (14.04%, 145/1033) and Hispanics (14.52%, 150/1033), which closely mirrors that of the US population (13.1% and 16.7%, respectively) [52]. The sample was highly motivated to quit (mean 8.5 out of a maximum of 10, where 10 equals the highest level of motivation), and had made an average of 3 quit attempts in the prior year.

**Table 1:** Baseline characteristics of study participants using the BecomeAnEX.org smoking cessation website (N=1033).

Demographic variables		n (%)	Mean (SE)
**Gender**			
	Female	534 (51.69)	
	Male	499 (48.31)	
**Race/ethnicity**			
	Non-Hispanic white	700 (67.76)	
	Non-Hispanic black	145 (14.04)	
	Hispanic	150 (14.52)	
	Other	38 (3.68)	
**Age, years**			
	18-24	120 (11.62)	
	25-44	531 (51.40)	
	45-64	351 (33.98)	
	≥65	31 (3.00)	
**Education**			
	Less than high school	62 (6.00)	
	High school diploma/GED	240 (23.23)	
	Some College	483 (46.76)	
	College degree or higher	248 (24.01)	
**Smoking variables**			
	Fagerstrom Test for Nicotine Dependence^a^		5.22 (0.07)
	Motivation to quit (0-10)		8.48 (0.07)
	Number of quit attempts in the past year		3.06 (0.20)
	Number of smoking peers (0-5)		2.05 (0.05)
	Has a partner who smokes	332 (32.14)	
	Household smoking	581 (56.24)	
	Advised to quit by a health care professional	847 (82.00)	
**Health status**			
	Excellent	72 (6.97)	
	Very good	304 (29.43)	
	Good	396 (38.33)	
	Fair	205 (19.85)	
	Poor	56 (5.42)	
**Psychological status**			
	Baseline sadness (1-6)^b^		3.45 (0.04)
	Cohen Perceived Stress Scale, baseline		7.51 (0.09)
**Baseline partner support** ^**c**^			
	Positive support		11.28 (0.10)
	Negative support		9.00 (0.12)
**Frequency of use of the Internet**			
	Several times a day	807 (78.12)	
	Approximately once a day	162 (15.68)	
	<5 days a week	64 (6.20)	

^a^ Range of responses for Fagerstrom Test was was 0-10.

^b^ Measured using the 12-Item Short Form Health Survey (SF-12) questionnaire.

^c^ Measured using the 6-item modified Partner Interaction Questionnaire (PIQ).

A comparison of study participants to other BecomeAnEX.org members that joined the site during the study period showed that study participants were more likely than non-study participants to smoke at least a pack of cigarettes a day (38.88% vs 25.57%, *P*<.001), more motivated to quit within the next 30 days (75.24% vs 66.10%, *P*<.001), and more likely to have made at least 1 quit attempt in the past year (64.88% vs 50.43%, *P*<.001) [data not shown].

### Website Utilization

The median for total website visits over the 6-month study period was 2 (IQR 1-4), the median number of total minutes on BecomeAnEX.org was 32 (IQR 17-57), and the median number of total pages accessed was 24 (IQR 12-60). Of the total sample, 40.27% (416/1033) visited the website once, 44.63% (461/1033) visited 2 to 5 times, and the remaining 15.10% (156/1033) visited more than 5 times. The quit plan was the most-accessed feature, with almost 90% (920/1033) of the sample accessing it by the end of the study period. Other commonly accessed features included the cigarette tracker, the Beat Your Smoking Triggers, the BecomeAnEX.org community, and the separation exercises. When asked whether BecomeAnEX.org was helpful in their efforts to quit smoking, almost 90% of study participants at each of the 3 follow-up periods said yes: 1 month=86.12% (614/713), 3 month=88.12% (549/623), and 6 month=90.47% (503/556). When asked which feature (apart from the overall My Quit Plan summary page) was most helpful, respondents were most likely to endorse the cigarette tracker (20.50%, 107/522), the separation exercises (23.56%, 123/522), and the BecomeAnEX.org community (14.75%, 77/522). The features deemed least helpful by respondents at the last follow-up included the quit smoking resources (2.68%, 14/522), the nicotine addiction videos (3.64%, 19/522), and the quit smoking support exercises (4.98%, 26/522). Data indicated that use of the features on BecomeAnEX.org occurred early upon registering because there was only minimal increases (<5%) in participants accessing new features beyond the initial 1-month period. Finally, although not recorded by Omniture SiteCatalyst, survey data showed that 10% of participants reported visiting the BecomeAnEX Facebook page, and 3% visited the BecomeAnEX Twitter account over the study period.

### Quit Rates

At 6 months, 7- and 30-day abstinence in the responder-only sample was 20.68% and 18.35%, respectively (see Table 2). Using ITT analysis, the rates of 7- and 30-day abstinence were 11.13% and 9.87%, respectively. Rates of abstinence increased dramatically as a function of website utilization: both 7- and 30-day abstinence rates were 10% or less for participants who had visited BecomeAnEX.org only 1 or 2 times, but jumped to 32.82% and 29.34% for those who had visited the website 3 or more times during the study period.

**Table 2:** Smoking behavior of survey responders over the course of the study (N=1033).

Quit rates		Baseline^a^	Follow-up		
			1 month n=713	3 months n=623	6 months n=556
**Quit attempts, %**					
	Yes	72.22	73.38	82.48	82.73
	No	27.78	26.62	17.52	17.27
Number of quit attempts, mean (SE)		3.06 (0.20)	2.12 (0.16)	3.24 (0.22)	3.52 (0.28)
**Responder quit rates, %**					
	7-day abstinence	n/a	13.18	18.14	20.68
	30-day abstinence	n/a	5.19	14.93	18.35
**Intent-to-treat quit rates** ^**b**^					
	7-day abstinence	n/a	9.10	10.94	11.13
	30-day abstinence	n/a	3.58	9.00	9.87

^a^ Smoking status at baseline is from BecomeAnEX.org registration data.

^b^ Intent-to-treat treats all nonresponders as current smokers.

### Characteristics of Respondents Abstinent at 6-months

Respondents abstinent for at least 7 days at the 6-month follow-up were more likely than those currently smoking to be non-Hispanic white (80.00% vs 62.36%, *P*<.001), less likely to be Hispanic (6.09% vs 23.36%, *P*<.001), less likely to have at least a college education (17.39% vs 31.29%, *P*=.003), less likely to have used the website only once (19.13% vs 41.04%, *P*<.001), and more likely to have accessed the BecomeAnEX.org community page (55.65% vs 35.37%, *P*<.001). Of respondents who accessed the BecomeAnEX.org community, those who were abstinent for at least 7 days at the 6-month follow-up, visited more often during the study period (mean 31.7, SE 12.7 vs mean 5.26, SE 2.62, *P*=.045] than those currently smoking at 6 months . There were no statistically significant differences by age or gender between those abstinent at the 6-month follow-up and those currently smoking.

### Generalized Estimating Equations Models: Website Utilization and Quit Outcomes

Results of the GEE regressions examining the association between website usage and quit behavior are displayed in Table 3. Although there was no statistically significant association between visits to the website and quit attempts, visits to the website was positively associated with both 7- and 30-day abstinence over time in adjusted analyses (both *P*<.001). When the most-accessed Web features were also put into the model (Model 2), visits to the website was still significantly associated with both 7-day (*P*<.001) and 30-day abstinence (*P*=.008). Accessing the cigarette tracker was negatively associated with quit attempts over time, such that those accessing this feature more often were less likely to make a quit attempt. Those who accessed the separation exercises were 2 or more times as likely to make a quit attempt (odds ratio [OR] 2.05, 95% CI 1.00-4.19, *P*=.05) as compared to those who did not access these exercises. For the outcome of 7-day abstinence, those who accessed the BecomeAnEX.org community once had an increased odds of 7-day abstinence (OR 1.74, 95% CI 1.13-2.67. *P*=.012), as compared to those who did not access the community. This was increased even further among those who accessed the BecomeAnEX.org community 2 or more times (OR 2.22, 95% CI 1.34-3.69, *P*=.002) Accessing the separation exercises once (OR 1.58, 95% CI 1.01-2.48, *P*=.045) or at least twice (OR 1.91, 95% CI, 1.00-3.65, *P*=.049) were also significantly related to 7-day abstinence. Increases in the odds of 30-day abstinence were only associated with assessing the BecomeAnEX.org community at least 2 times (OR 2.42, 95% CI 1.35-4.34, *P*=.003). However, the association between the separation exercises and abstinence remained borderline significant.

**Table 3:** Association between website usage and quit behavior over time using generalized estimating equations (GEE).

Regression models		Quit attempts		7-Day abstinence		30-Day abstinence	
		OR (95% CI)	*P*	OR (95% CI)	*P*	OR (95% CI)	*P*
**Model 1** ^**a**^							
	Visits to the website^b^	1.09 (0.94-1.28)	.26	2.04 (1.75-2.39)	<.001	1.73 (1.47-2.05)	<.001
**Model 2** ^**a**^							
	Visits to the website^b^	1.10 (0.88-1.38)	.39	1.55 (1.26-1.91)	<.001	1.36 (1.08-1.70)	.008
**Use of Community feature**							
	1 vs 0 times	0.79 (0.53-1.17)	.24	1.74 (1.13-2.67)	.01	1.37 (0.81-2.30)	.24
	≥2 vs 0 times	1.06 (0.62-1.83)	.82	2.22 (1.34-3.69)	.002	2.42 (1.35-4.34)	.003
**Use of Cigarette Tracker feature**							
	1 vs 0 times	0.62 (0.40-0.96)	.03	1.27 (0.48-1.21)	.33	0.97 (0.56-1.70)	.30
	≥2 vs 0 times	0.42 (0.22-0.80)	.01	0.82 (0.28-1.01)	.55	0.81 (0.38-1.72)	.13
**Use of Beat Triggers exercise feature**							
	1 vs 0 times	1.36 (0.87 2.14)	.18	1.16 (0.72-1.88)	.54	0.87 (0.50-1.52)	.63
	≥2 vs 0 times	1.20 (0.60-2.37)	.61	1.20 (0.61-2.36)	.60	0.86 (0.39-1.88)	.70
**Use of Separation exercise feature**							
	1 vs 0 times	0.99 (0.65-1.53)	.97	1.58 (1.01-2.48)	.05	1.67 (0.98-2.83)	.06
	≥2 vs 0 times	2.05 (1.00-4.19)	.05	1.91(1.00-3.65)	.05	1.99 (0.94-4.22)	.07

^a^ All models adjusted for demographics, nicotine dependence, baseline quit attempts, peer smoking, household smoking, motivation to quit, positive and negative social support, health status, advice to quit from a health care provider, use of at least one cessation aide, baseline depression, baseline perceived stress, having a partner who smokes, and frequency of use of the Internet. Model 1 includes all covariates plus visits to the website. Model 2 includes all covariates, visits to the website, and use of specific BecomeAnEX.org features.

^b^ Represented as the log of total visits to the BecomeAnEX.org website over the study period.

### Predictors of Website Utilization

The final analysis examined the predictors of website usage determined in the previous models to be associated with quit behavior, namely visits to the website and use of the BecomeAnEX.org community and separation exercises (Table 4). Data show that there were few predictors of website visits. Visits were less likely among Hispanics (beta coefficient=–0.18, SE=0.08, *P*=.03) as compared to non-Hispanic whites, and increased as function of motivation to quit (beta coefficient=0.03, SE=0.01, *P*=.02). Accessing the BecomeAnEX.org community was predicted by higher education, positive partner support at baseline (OR 1.06, 95% CI 1.01-1.10, *P*=.02), and motivation to quit (OR 1.26, 95% CI 1.16-1.37, *P*<.001). Higher reported stress at baseline was also associated with accessing the community (OR 1.05, 95% CI 1.00-1.11, *P*=.48). Negative partner support at baseline decreased the probability of accessing the community (OR 0.91, 95% CI 0.88-0.95, *P*<.001). There were many predictors of accessing the separation exercises. Separation exercises were more likely to be accessed in respondents 18 to 24 years of age, those with higher education, and those with increased motivation to quit (OR 1.37, 95% CI 1.25-1.49, *P*<.001), but less likely in non-Hispanic blacks (OR 0.37, 95% CI 0.25-0.56, *P*<.001) and those of other race/ethnicities (OR 0.42, 95% CI 0.20-0.86, *P*=.02) as compared with non-Hispanic whites and those with negative partner support at baseline (OR 0.96, 95% CI 0.93-1.00, *P*=.29). Those who had made at least 1 quit attempt (OR 0.73, 95% CI 0.54-1.00, *P*=.05) were also less likely to access the separation exercises, although this was of borderline significance.

### Weighted Analyses

The 7- and 30-day weighted abstinence rates over the study period were almost identical to the unweighted rates, at 21.83% and 17.68%, respectively. Results were also similar on the measures regarding helpfulness of the website, use of Web features, and smoking behavior (see Multimedia Appendix 1). The first GEE regression model (Model 1) examining the association between website utilization and quit behavior was similar to the unweighted analyses. However, there were several differences in Model 2. Although the association between visits to the website and both 7-day abstinence (OR 1.75, 95% CI 1.35-2.28, *P*<.001) and 30-day abstinence (OR 1.54, 95% CI 1.17-2.02, *P*=.002) was similar, there was no longer a statistically significant relationship between any of the website features and quit behavior (see Multimedia Appendix 1).

**Table 4:** Demographic characteristics and smoking behavior with use of BecomeAnEX.org website and its associated features (unweighted).

Demographic variables		Visits to website		Use of the community		Separation exercises	
		Beta coefficient (SE)	*P*	OR (95% CI)	*P*	OR (95% CI)	*P*
Gender							
	Male	–0.3 (0.05)	.56	0.88 (0.68-1.15)	.36	0.75 (0.57-0.97)	.03
	Female	REF		REF		REF	
Race/ethnicity							
	Non-Hispanic white	REF		REF		REF	
	Non-Hispanic black	–0.11 (0.07)	.13	0.84 (0.58-1.22)	.37	0.37 (0.25-0.56)	<.001
	Hispanic	–0.18 (0.08)	.03	0.72 (0.44-1.19)	.20	0.74 (0.46-1.20)	.22
	Other	–0.02 (0.13)	.85	0.59 (0.28-1.22)	.16	0.42 (0.20-0.86)	.02
Age, years							
	18-24	REF		REF		REF	
	25-44	–0.13 (0.08)	.10	0.86 (0.56-1.32)	.48	0.56 (0.37-0.84)	.005
	45-64	–0.08 (0.08)	.32	1.41 (0.90-2.22)	.14	0.51 (0.32-0.79)	.002
	≥65	–0.22 (0.15)	.15	0.79 (0.34-1.84)	.58	0.35 (0.15-0.84)	.02
Education							
	Less than high school	–0.04 (0.10)	.69	1.47 (0.82-2.62)	.20	0.32 (0.15-0.68)	.003
	High school diploma/GED	REF		REF		REF	
	Some College	0.07 (0.06)	.20	1.51 (1.09-2.10)	.01	1.49 (1.08-2.06)	.02
	College degree or more	0.10 (0.07)	.17	1.59 (1.07-2.36)	.02	1.80 (1.22-2.65)	.003
Baseline smoking variables							
	Past-year quit attempts	–0.09 (0.06)	.10	0.82 (0.60-1.10)	.18	0.73 (0.54-1.00)	.05
	Fagerstrom Test for Nicotine Dependence	0.02 (0.02)	.46	1.05 (0.94-1.18)	.36	0.90 (0.80-1.00)	.06
	Motivation to quit (1-10)	0.03 (0.01)	.02	1.26 (1.16-1.37)	<.001	1.37 (1.25-1.49)	<.001
	Use of at least one cessation aide	0.01 (0.05)	.90	0.89 (0.68-1.17)	.40	1.16 (0.88-1.52)	.29
Health status							
	Excellent	0.01 (0.14)	.93	1.16 (0.51-2.64)	.73	0.46 (0.20-1.04)	.06
	Very good	0.06 (0.11)	.57	1.93 (1.02-3.66)	.04	0.90 (0.49-1.68)	.74
	Good	0.08 (0.11)	.48	1.62 (0.88-2.97)	.12	0.91 (0.51-1.65)	.77
	Fair	0.07 (0.11)	.56	1.54 (0.83-2.88)	.98	0.80 (0.43-1.47)	.47
	Poor	REF		REF		REF	
Advised to quit from a health care professional		0.04 (0.06)	.51	1.33 (0.91-1.93)	.14	0.90 (0.63-1.29)	.58
	Baseline sadness	–0.02 (0.02)	.34	1.00 (0.88-1.13)	.98	0.94 (0.83-1.07)	.34
	Baseline perceived stress	0.01 (0.01)	.25	1.05 (1.00-1.11)	.05	1.01 (0.96-1.07)	.69
Baseline partner support (PIQ)							
	Positive support	0.002 (0.01)	.83	1.06 (1.01-1.10)	.02	1.02 (0.97-1.06)	.48
	Negative support	–0.008 (0.01)	.22	0.91 (0.88-0.95)	<.001	0.96 (0.93-1.00)	.03
Peer smoking		-0.01 (0.01)	.59	1.03 (0.95-1.11)	.55	1.04 (0.96-1.13)	.32
	Has a partner who smokes	–0.08 (0.05)	.89	0.85 (0.63-1.14)	.27	0.95 (0.71-1.28)	.76
	Household smoking	–0.04 (0.05)	.45	1.04 (0.79-1.37)	.80	0.87 (0.66-1.14)	.32
Frequency of use of the Internet^a^		–0.03 (0.03)	.33	0.92 (0.76-1.12)	.42	1.16 (0.96-1.40)	.12

^a^ Modeled as a continuous variable, the higher the number, the less often they use.

## Discussion

This observational prospective study adds to the growing body of literature examining the effectiveness of an interactive Web-based smoking cessation intervention in promoting quit behavior and presents new information on factors predicting general utilization and use of specific features of this website. Abstinence rates were approximately 20.68% for 7-day and 18.35% for 30-day abstinence at 6 months in both weighted and unweighted analyses, and 11.13% and 9.87% for 7- and 30-day abstinence in the ITT analysis. These rates were similar to quit rates achieved in other Web-based smoking cessation interventions [18] and highlight the importance of finding ways to increase repeat visits, engagement, and retention of new users, both in the general population and those participants involved in a real-world evaluation study of a Web-based intervention.

Adjusting for a host of demographic factors, smoking history, and psychosocial variables thought to potentially influence quit behavior, the number of visits to BecomeAnEX.org significantly predicted both 7- and 30-day abstinence. Even after accounting for the independent and statistically significant effects of accessing the BecomeAnEX.org community and separation exercises, number of visits still predicted abstinence. Motivation to quit was a key predictor of visiting the website and accessing both the community and the separation exercises, whereas negative partner support decreased the likelihood of accessing both of these features.

Results presented here are in accordance with several studies that have reported significant associations between number of visits to a Web-based smoking cessation intervention and abstinence [28-32] and build on existing evidence suggesting the importance of interactive features in promoting cessation [18,53] as well as other behavioral outcomes.[54-56] Among the top features accessed on the website, the BecomeAnEX.org community most significantly predicted abstinence in the sample, followed by the separation exercises. The remaining interactive features—the Cigarette Tracker, Beat Your Triggers exercises, and the Set a Quit Date feature—did not promote abstinence. In fact, accessing the cigarette tracker was negatively associated with quit attempts, potentially because participants accessing this feature were still contemplating and/or preparing for a behavior change and not ready to quit. It may work, therefore, to move someone down the pathway toward behavior change and/or it may be that other elements are needed in this feature to prompt a smoker to quit. These findings suggest that interactivity itself may not be sufficient to change behavior. The challenge is to identify how features such as the community and separation exercises work to promote abstinence, to consider whether such features successfully help move smokers at all stages of behavior change toward making a quit attempt, and then design Web-based interventions that capitalize on this information.

Data presented here also suggest that the results achieved in a study sample may differ from the broader population of registrants.Although most of the weighted results were similar to the unweighted results, associations between BecomeAnEX.org community utilization and abstinence and between utilization of the separation exercises and abstinence were in the same direction, but the effects were not statistically significant in weighted analyses. This may be because of underlying differences in motivation to quit between general registrants and a sample that must go through multiple steps of recruitment before entering into a study. It could also reflect differences in social support between registrants and the sample that influenced the use of the interactive features and potentially could influence how these features promoted cessation.

Maximizing the effectiveness of any cessation intervention is dependent on reaching the target audience, but also engaging and motivating the smoker to take concrete steps toward quitting. Data from this study and others suggest that engagement may be the key driver of success, but the nature of engagement and the mechanisms underlying the effectiveness of engagement in promoting abstinence may differ across subgroups. For example, data presented here show that those abstinent at 6 months were more likely to be white, less likely to be Hispanic, and less likely to be at least college-educated. The reasons for this are unclear, but may be because of the layout, design, language, and features embedded in the quit program. Further research is needed to clarify why certain populations appear to benefit more than others from this and other Web-based smoking cessation interventions. Because one intervention will never appropriately cater to the needs of all subpopulations, it is essential for nonprofit, for-profit, and governmental groups to collaboratively develop and promote complementary and synergistic programs to maximize effectiveness across the population as a whole.

There are several limitations to this study. First, results are limited to those respondents who completed at least 1 follow-up survey. Response rates on the follow-up surveys (69.41%, 60.31%, and 53.92% at 1, 3, and 6 months, respectively) were as good or better than those recorded for many Web-based studies [20,26,27,29,32,57] (eg, 39.4% online and 38.2% by telephone [58] and 48% and 45% at 3 and 6 months [59]). Although GEE was used to maximize the information obtained from the prospective data, these data support the need to employ techniques to minimize attrition to provide the most valid estimates of the intervention’s effectiveness [60-62]. Second, although software tracked respondents’ activity online, data presented here are limited to what features the respondent accessed and do not necessarily indicate full utilization of a specific feature of the site. For example, they could have accessed a page on the BecomeAnEX.org community but not posted or responded to a thread. Follow-up studies will need to address this further by using more in-depth tracking metrics. Third, individual-level utilization of the mobile BecomeAnEX.org site could not be characterized by using the Omniture SiteCatalyst software. Since the mobile site was not heavily promoted or used during the trial, however, it is unlikely to have significantly biased results. Fourth, it is possible that the weighting scheme did not entirely correct for differences in website utilization, motivation to quit, or smoking behaviors between the study sample and the larger BecomeAnEX.org population. However, the demographic variables used for weighting were shown to be significantly correlated with motivation to quit, quit attempts, cigarettes per day, and website utilization, thereby helping to correct any differences that exist between these populations. Fifth, the study period was only 6 months and outcomes may have changed if respondents were followed for a longer period of time. Six-month follow-up for this type of observational study is standard in the literature [18,37]. Sixth, there was no control group used in this study, so the overall effectiveness versus placebo could not be determined. Finally, differences between the study sample and the larger population of BecomeAnEX.org members may limit generalizability of the results. These challenges to generalizability confront any real-world study that focuses on practical trials from an implementation-dissemination research methods perspective [38,40].

Tobacco use causes nearly 6 million deaths and costs hundreds of billions of dollars worldwide each year [63]. Interventions are needed that are low cost, accessible, sustainable, and can reach large populations [64]. This study adds to the growing literature that seeks to understand how to maximize the effectiveness of Web-based smoking cessation interventions. It is the first observational study that weights the data to obtain representative estimates of the larger population of website registrants, thereby addressing a key question of the effectiveness of Web-based smoking cessation interventions in the greater population of users. This study also employs website tracking software to generate reliable estimates of website utilization and also controls for many potential confounders that are not often considered in such analyses, including stress, depression, and both positive and negative partner support. Results suggest that the primary driver of success in Web-based smoking cessation interventions is engagement through multiple visits, and involvement in the community and other interactive features. Findings emphasize the importance of increasing the interaction of new users with other smokers wanting to quit and finding ways to increase repeat visits and retention. This study also addresses a key scientific question of the type of smoker most likely to use and benefit from Web-based smoking cessation interventions. Data suggest that public health organizations looking to affect the greatest change in behavior may achieve maximal results by marketing these resources to smokers who are highly motivated to quit. Future research should further examine the variation in effectiveness across demographic subgroups, how use of interactive features promotes cessation, mechanisms underlying effectiveness of these features, whether there are synergies among different components of the website, and how results gained through evaluations of Web-based cessation interventions apply to the greater population of registered users. This information can help advance the development and use of Web-based smoking cessation interventions for the greatest impact on population-level smoking rates.

## Multimedia Appendix 1

Weighted analysis tables.

## Abbreviations

GED: Generalized Education Development Test
GEE: generalized estimating equations
IQR: interquartile range
ITT: intent-to-treat
IRB: Institutional Review Board
OR: odds ratio
PIQ: Partner Interaction Questionnaire
SF-12: 12-Item Short Form Health Survey
