# Supplementary material for: Engagement Promotes Abstinence in a Web-based Cessation Intervention: Cohort Study
Source: J Med Internet Res. 2013 Jan 28;15(1):e14. doi: 10.2196/jmir.2277 (PMC3636070; doi:10.2196/jmir.2277)
Supplement: Supplementary file 1 [file jmir_v15i1e14_app1.pdf]

APPENDIX TABLES

Table 1. Smoking behavior in survey responders over the course of the study using weighted data

	**Baseline**	**30-day follow-up**	**90-day follow-up**	**180-day follow-up**
	% (N)	***% (N)***	***% (N)***	***% (N)***
**Quit attempts**				
Yes	72.9%	75.6%	80.2%	78.9%
No	27.1%	24.4%	19.8%	21.1%
**Number of quit attempts**				
Mean (SE)	2.92 (0.22)	2.06 (0.22)	3.09 (0.28)	2.97 (0.34)
**7-day abstinence**	n/a	10.4%	14.8%	21.8%
**30-day abstinence**	n/a	3.0%	13.0%	17.7%
**ITT 7-day abstinence***	n/a	6.7%	8.4%	10.4%
**ITT 30-day abstinence**	n/a	2.0%	7.4%	8.5%

*The weighting is an additional adjustment to the Intent-to-treat adjustment, which treats all non-responders as current smokers, so may not be a valid rate.

Table 2. Association between website usage and quit behavior over time using GEE in weighted data

	**Quit Attempts**		**7-Day Abstinence**		**30-Day Abstinence**	
	aOR [95% CI]	***p-value***	***aOR [95% CI]***	***p-value*****	***aOR [95% CI]***	***p-value***
**Model 1[1]**						
**Visits to the website***	1.11 (0.88, 1.40)	0.38	1.98 (1.56, 2.50)	0.00	1.76 (1.43, 2.16)	0.00
**Model 2[1]**						
**Visits to the website***	1.22 (0.81, 1.84)	0.34	1.75 (1.35, 2.28)	0.00	1.54 (1.17, 2.02)	0.00
**Use of web features**						
EX Community						
1 vs. 0 times	0.51 (0.28, 0.94)	0.03	2.03 (0.96, 4.29)	0.06	1.66 (0.81, 3.42)	0.17
2+ vs. 0 times	0.67 (0.24, 1.85)	0.44	1.52 (0.68, 3.44)	0.31	2.07 (0.88, 4.88)	0.10
Cigarette Tracker						
1 vs. 0 times	0.63 (0.31, 1.27)	0.19	1.16 (0.52, 2.61)	0.72	1.02 (0.48, 2.16)	0.96
2+ vs. 0 times	0.49 (0.20, 1.22)	0.12	0.95 (0.37, 2.44)	0.91	1.17 (0.41, 3.33)	0.77
Beat Triggers Exercise						

1 vs. 0 times	1.35 (0.66, 2.75)	0.41	0.88 (0.38, 2.04)	0.77	0.60 (0.28, 1.29)	0.19
2+ vs. 0 times	1.01 (0.31, 3.32)	0.99	1.25 (0.42, 3.69)	0.69	1.15 (0.40, 3.31)	0.79
Separation Exercise						
1 vs. 0 times	1.33 (0.63, 2.81)	0.46	1.43 (0.65, 3.18)	0.38	1.64 (0.79, 3.40)	0.19
2+ vs. 0 times	1.94 (0.59, 6.36)	0.27	1.17 (0.39, 3.48)	0.78	0.86 (0.27, 2.73)	0.80

*Represented as the log of total visits to the becomanex.org website over the study period
**P<0.01. Details on exact p-value in text.
